# Repulsive Guidance Molecule-a and Central Nervous System Diseases

**DOI:** 10.1155/2021/5532116

**Published:** 2021-05-04

**Authors:** Jinhua Tang, Xiaopeng Zeng, Hang Li, Lu Ju, Jinzhou Feng, Jun Yang

**Affiliations:** Department of Neurology, The First Affiliated Hospital of Chongqing Medical University, Chongqing 400016, China

## Abstract

Repulsive guidance molecule-a (RGMa) is a member of glycosylphosphatidylinositol- (GPI-) anchored protein family, which has axon guidance function and is widely involved in the development and pathological processes of the central nervous system (CNS). On the one hand, the binding of RGMa and its receptor Neogenin can regulate axonal guidance, differentiation of neural stem cells into neurons, and the survival of these cells; on the other hand, RGMa can inhibit functional recovery of CNS by inhibiting axonal growth. A number of studies have shown that RGMa may be involved in the pathogenesis of CNS diseases, such as multiple sclerosis, neuromyelitis optica spectrum diseases, cerebral infarction, spinal cord injury, Parkinson's disease, and epilepsy. Targeting RGMa can enhance the functional recovery of CNS, so it may become a promising target for the treatment of CNS diseases. This article will comprehensively review the research progression of RGMa in various CNS diseases up to date.

## 1. Introduction

RGMa (repulsive guidance molecule-a) is a member of glycosylphosphatidylinositol (GPI)-anchored protein family [1]. It was first found in the visual system of chicken embryo, with axon guidance function [1]. Three members of the RGM family, RGMa, RGMb, and RGMc were found in vertebrates [2]. Their 3D structures have been partially discovered [2, 3]. RGMa and RGMb are expressed in the central nervous system (CNS) and other tissues (heart, lung, liver, small intestine, etc.) with a nonoverlapping form, while RGMc is only expressed in the skeletal muscle, liver, and blood [2]. The RGMa gene is located on chromosome 15q26.1 and encodes a protein of 450 amino acids [1, 2]. RGMa consists of GPI-anchored C-terminal signal peptide, N-terminal signal peptide, and RGD motif (only found in RGMa and RGMc) and partial von Willebrand factor type D [2, 4, 5]. RGMa exists in CNS such as neural stem cells, neuron cells, and myelin sheath in both soluble and membrane-bound forms [4, 5]. It binds with type I transmembrane protein Neogenin and plays the biological functions of axon guidance and neuron survival through the FAK-RhoA signaling pathway [4, 5]. In addition, as a coreceptor of bone morphogenetic proteins (BMPs), RGMa can bind to BMP-2, BMP-4, and other BMP family molecules and participate in iron metabolism, bone development, and axon regeneration through the BMP-BMPR signaling pathway [2, 5]. Currently, a number of studies have shown that RGMa is highly expressed in the injured lesions in patients with multiple sclerosis, neuromyelitis optica spectrum diseases, cerebral infarction, spinal cord injury, and Parkinson's disease [6–11]. It has also been reported that it can promote the functional recovery of the nervous system by inhibiting RGMa [6–11]. However, the expression of RGMa is low in patients with epilepsy [12]. Upregulation of RGMa can reduce epileptic seizures [12]. These data indicated that RGMa may be involved in the pathogenesis of the above diseases and may become a potential target for the treatment of CNS diseases.

## 2. Role of RGMa in CNS Physiology

### 2.1. Cell Proliferation and Differentiation

Both RGMa and Neogenin are highly expressed in intestinal neural stem cells during proliferation and differentiation [13]. The loss of RGMa and Neogenin resulted in the decrease of neurons, glial cells, and ganglia in the intestinal system, indicating that RGMa is involved in the proliferation and differentiation of intestinal neural stem cells [13]. RGMa had a rejection effect on differentiating progenitors via Neogenin [13, 14]. In the midbrain of a chicken embryo, RGMa overexpression can temporarily inhibit cell proliferation [15]. In addition, RGMa promotes neuronal differentiation in the midbrain through Neogenin signal transduction [15].

### 2.2. Cell Adhesion and Migration

**Figure 1:**
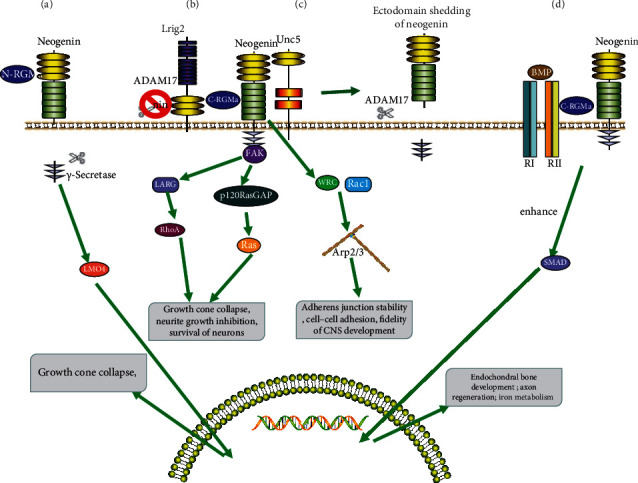
Mechanisms for RGMa Signal Transduction. (a) The role of N-RGM depends on the release of Neogenin intracellular domain by *γ*-secretase and LMO4. It has been suggested that Neogenin intracellular domain may enter the nucleus together with LMO4 and regulates gene transcription and growth cone collapse. (b) In general, C-RGM-Neogenin binding can activate RhoA through Unc5 and LARG and inactivate Ras through FAK and p120 RasGAP, thus inducing growth cone collapse and playing the role of axon guidance and regulation of neuronal survival. The binding of C-RGMa with Neogenin inhibits the interaction between Lrig2 and Neogenin. At this time, ADAM17 can cleave Neogenin and cause the extracellular domain of Neogenin to fall off, eventually leading to signal termination. Therefore, LRIG2 and ADAM17 can regulate the sensitivity of neurons to RGMa. (c) In epithelial cells, Neogenin binds to and localizes the wave regulatory complex (WRC), leading to actin nucleation via Arp2/3, which also requires Rac1 to activate the stability of adhesion junctions. (d) RGMa acts as a coreceptor of bone morphogenetic protein (BMP) and has been proposed as a structural bridge between BMP and Neogenin. A recently proposed model suggests that RGMa induces endocytosis of BMP receptor complexes, thereby activating classical Smad signaling. The interaction between RGM and BMP signal transduction has been involved in iron metabolism, bone development, axon regeneration, and so on.

RGMa can improve the adhesion of embryonic cells in vitro, and RGMa overexpression can also induce the migration defects in early embryonic ectodermal cells, indicating that RGMa is related to the adhesion and migration of embryonic cells [16]. RGMa increases the adhesion between cells through Neogenin, recruiting cell adhesion molecules [17]. Moreover, RGMa and Neogenin jointly act on adhesion junctions (AJ) to regulate actin and maintain epithelial fidelity. In epithelial cells, Neogenin binds to and localizes the wave regulatory complex (WRC), leading to actin nucleation via Arp2/3 (Figure 1(c)) [2]. RGMa induces cell migration through Neogenin, independent of the BMP pathway [17]. Recent studies have shown that Neogenin-Netrin-1-RGMa complex regulates neuron migration [18]. The vWF and RGD domains in RGMa play a functional role in cell adhesion and cell migration [17].

### 2.3. Neurogenesis and Neural Tube Closure

RGMa and Neogenin can coregulate the differentiation and migration of embryonic neurogenesis, and they can also coregulate the development of an adult central nervous system (Figures 1(a) and 1(b)) [15, 17]. In addition, RGMa can induce Neogenin protein hydrolysis and promote neural tube morphogenesis [19]. The closure defects of neural tube may occur if RGMa is exhausted [19]. Neogenin-Netrin-1-RGMa complex may regulate neurogenesis and neural tube closure through the RhoA/ROCK pathway [18]. In addition, whether the RGMa-BMP pathway plays a role in neurogenesis is a hot topic in future research [17].

## 3. Role of RGMa in CNS Pathology

### 3.1. Survival of Neurons

On the one hand, inhibition of RGMa in adult dentate gyrus can increase the number of new neurons; on the other hand, inhibition of Neogenin can improve neuron survival and behavioral recovery after spinal cord injury [20, 21]. It has been proved that both RGMa and Neogenin regulate the survival of neurons [20, 21]. Some scholars believe that RGMa binds to Neogenin on neural stem cells and regulates neuron survival by regulating caspase-3 and Rock [15, 17]. Other scholars believe that the combination of RGMa and Neogenin affects the survival of neurons in the CNS through death-associated protein kinase (DAPK) and LMO4. DAPK affects cell survival by activating the apoptotic pathway, while LMO4 affects the cytoskeleton and gene expression [2, 22].

### 3.2. Synapse Formation

RGMa can inhibit synapse formation by interfering with the expression of presynaptic protein synapsin-1 and postsynaptic protein PSD-95 in cortical neurons [23]. Inhibition of RGMa can increase the coexpression of the above two proteins, thus enhancing the synaptic formation after spinal cord injury [23]. RGMa, especially C-RGMa, may inhibit synaptic formation through Neogenin [24]. The latest research shows that RGMa can regulate neuronal branching through the RhoA pathway to mediate synaptic plasticity [25].

### 3.3. Growth Cone Collapse and Axon Growth Inhibition

RGMa can inhibit the axon growth after CNS injury, but the specific mechanism is not clear. It may inhibit the axon growth by stimulating neurons to induce RhoA and ROCK (Rho-associated coiled-coil protein kinase) expression [5]. It has been confirmed that RGMa may cause cone collapse by activating downstream Rho-GTPase activity [26]. In addition, RGMa can regulate the phosphorylation of collapse response mediator protein-2 (CRMP-2) by activating Rho-kinase and glycogen synthase kinase 3 *β* (GSK-3*β*) signaling pathways, thus regulating the axonal shortening [26]. Two independent c-RGMa and n-RGMa can activate different intracellular pathways to regulate neuronal survival: (1) in general, c-RGMa combines with Neogenin to activate RhoA through Unc5 and LARG (Figure 1(b)) [27–29]. C-RGMa can inhibit axon growth through the Rho-GEF (LARG)/Rho/ROCK signaling pathway and also inactivate Ras through FAK and p120 Ras-GAP to induce growth collapse (Figure 1(b)) [27–29]. (2) When C-RGMa and Neogenin bind to inhibit the interaction between LRIG2 and Neogenin, then, C-RGMa can promote ADAM17 specific cleavage of Neogenin, resulting in signal termination (Figure 1(b)) [27–29]. (3) N-RGMa mainly depends on *γ*-secretase to cleave the intracellular part of Neogenin to generate intracellular domain [27]. This domain can inhibit axonal growth by binding to LIM protein 4 (LMO4) (Figure 1(a)) [27]. Inhibition of RGMa with specific antibodies can promote axonal germination, regeneration, and motor recovery after spinal cord injury (SCI) in primates [30].

### 3.4. Immunoregulation

RGMa can regulate T cell activation and autoimmunity through dendritic cells (DCS) [31]. RGMa in dendritic cells can also bind to Neogenin on CD4^+^ T cells to activate inflammatory cells, enhance the adhesion between inflammatory cells and ICAM-1, and indirectly regulate the release and diffusion of cytokines [31]. Treatment with RGMa neutralizing antibody can cause dendritic cell tolerance and immunomodulatory function; reduce the levels of MHC II, CD86, CD80, and CD40; decrease the levels of IL-12, IL-1 *β*, and TNF-*α*; and increase the secretion of IL-10, resulting in reducing T cell proliferation and enhancing the T cell differentiation into Th2 cells [32].

### 3.5. Inhibition of Angiogenesis

RGMa is a negative regulator of angiogenesis [33]. The binding of recombinant RGMa with Neogenin on endothelial cells can significantly reduce endothelial cell proliferation, migration, and formation of vascular endothelium, as well as the level of phosphorylated focal adhesion kinase (p-FAK Tyr397) [5, 33, 34]. In addition, F-actin assembled in the cytoskeleton was also significantly inhibited, thereby inhibiting cytoskeleton reorganization [5]. Removal of Neogenin or Unc5b could significantly reduce the effect of RGMa [5, 34]. RGMa can inhibit angiogenesis by down-regulating VEGF and p-FAK (Tyr397) in vitro [34, 35]. Recombinant RGMa can also inhibit angiogenesis [36].

## 4. RGMa as a Therapeutic Target in CNS Disorders

### 4.1. Multiple Sclerosis (MS)

Many researches have shown that RGMa plays an important role in MS. Demicheva et al. reported that the expression of RGMa was significantly increased in acute and chronic damaged plaques and normal white matter of CNS in MS patients [22]. The level of RGMa in the baseline blood was negatively correlated with the changes of Expanded Disability Status Scale (EDSS) in MS patients, indicating that the level of RGMa was closely related to neurological function [2, 6, 37]. The possible pathogeneses of RGMa in MS include the following:

1. Abnormal signal transduction of immune cells: since IL-17-expressing CD4^+^ T cells (Th17 cells) strongly expressed RGMa, so the combination of RGMa and Neogenin on immune cells can enhance the immune cell adhesion, promote their invasion to the brain, and enhance T cell response [5, 37]. Neutralizing RGMa antibody can reduce the severity of experimental autoimmune encephalomyelitis (EAE) in the MS animal model; secondly, it can inhibit peripheral blood T cell proliferation, block the production of inflammatory cytokines such as IL-2, IFN-*γ*, IL-17, and IL-4, and significantly reduce the level of CNS inflammatory cytokines in MS patients [5, 37].
2. Promotion of demyelinating production: in EAE, RGMa promotes the demyelination of CNS by enhancing the activation of CD4^+^ T cells [38, 39]. Our previous work also found that the usage of RGMa neutralizing antibody can reduce the demyelination level of EAE mice, thereby inhibiting the neurological damage.
3. Promotion of neurodegeneration: RGMa has strong inhibitory activity on axon regeneration and also plays a role in MS neurodegeneration [40]. The possible mechanism involves RGMa inducing Akt dephosphorylation in neurons by binding to Neogenin on Th17 cells [40]. Neutralizing RGMa antibody can enhance the axonal regeneration ability of inflammatory lesions, reduce axonal degeneration and clinical severity, and promote the growth of corticospinal tract and motor recovery in EAE mice [5, 7, 22, 40].
4. Inhibition of angiogenesis: angiogenesis is another key factor involved in the pathophysiology of EAE [41]. RGMa can inhibit the formation of endothelial vessels [5, 41].
5. Alter the permeability of blood-brain barrier (BBB): the damage of BBB is an important pathological feature of MS [42, 43]. Studies have found that the level of RGMa in cerebrospinal fluid in patients with triamcinolone acetonide treatment (its pharmacological effect is mainly on improving the BBB permeability) is reduced, which suggested that RGMa may be involved in the pathology of MS by regulating BBB permeability in MS patients (Table 1) [44, 45].

### 4.2. Neuromyelitis Optica Spectrum Disorders (NMOSD)

Systemic administration of anti RGMa antibody can delay the onset time, alleviate its clinical symptoms, and reduce inflammatory cell infiltration and axon damage in NMOSD rat model, indicating that inhibiting RGMa can effectively treat NMOSD [8]. The possible pathogeneses of RGMa in NMOSD include the following: (1) the loss of aquaporin-4 (AQP4) and glial fibrillary acid protein (GFAP) often occurred before the demyelination of NMOSD [46]. Anti-RGMa antibody could partially restore the expression of AQP4 and GFAP in NMOSD rats, resulting in preventing astrocytopenia and relieving clinical symptoms [8, 46]. (2) Anti-RGMa antibody can reduce the immune response of NMOSD rats, which may help to delay the attack and/or progress of NMOSD in the NMOSD rat model by reducing the number of activated microglia and reducing the infiltration of IL-17A ^+^ T cells [8, 47]. (3) Axonal injury is an early pathological feature of NMOSD, which can cause dyskinesia [40]. The treatment of anti RGMa antibody can reduce axonal degeneration and injury [8, 40, 48]. (4) Inhibition of RGMa can promote the repair of damaged neural network and delay the secondary progression of NMOSD (Table 1) [8, 49].

### 4.3. Cerebral Infarction

Our previous study found that an adenovirus vector can reduce BBB dysfunction in rats with middle cerebral artery occlusion (MCAO)/reperfusion by inducing specific RGMa silencing [9]. The possible mechanism was that RGMa participates in BBB injury through the CDC-42/PAK-1 pathway (Table 1) [9, 50]. We also found that RGMa can inhibit axonal growth by phosphorylating CRMP-2 through the Rho kinase and GSK-3*β* signaling pathways (Table 1) [51, 52]. Both RGMa and Neogenin were expressed in neurons and vessel endothelial cells after ischemia/reperfusion injury in rats, and angiogenesis, coupled with functional recovery, was enhanced after RGMa RNA interference against RGMa [34, 35]. The mechanism may lie in RGMa inhibiting angiogenesis through VEGF, Ang2, Ang1, and BDNF (Table 1) [34, 35]. In addition, the increased RGMa in patients with MCAO may be related to leptomeningeal collateral damage, which can predict the pathological state of leptomeningeal collateral by measuring the expression of RGMa mRNA in the early stage of stroke (Table 1) [53].

### 4.4. Spinal Cord Injury (SCI)

The treatment of spinal cord injury with anti-RGMa antibody can promote the recovery of hand agility and muscle strength [10, 30]. Possibly because the inhibition of RGMa promotes the survival and regeneration of neurons, it promotes the regeneration, repairs plasticity of corticospinal tract axons, improves motor function and gait recovery, and reduces nerve pain by reducing activated microglia (Table 1) [21].

### 4.5. Parkinson's Disease (PD)

RGMa is upregulated in the substantia nigra of Parkinson's disease patients [11]. RGMa can induce neuropathological and behavioral changes similar to Parkinson's disease [11]. If RGMa in substantia nigra dopaminergic (DA) neurons of Parkinson's disease mouse is significantly increased, it can lead to progressive dyskinesia, including motor coordination and imbalance, which is a typical manifestation of DA reduction in striatum [11, 54–56]. The mechanism may be the selective degeneration of DA neurons and the activation of microglia and astrocytes in substantia nigra induced by elevated RGMa (Table 1) [11, 57]. These data suggested that RGMa dysfunction plays an important role in Parkinson's disease [11, 57].

### 4.6. Epilepsy

RGMa has been considered a potential therapeutic agent for epilepsy [12, 58]. Some studies found that the levels of RGMa are significantly decreased in both temporal lobe epilepsy patients and experimental rats [12, 58]. Some studies also confirmed that overexpression of RGMa can inhibit epileptic seizures [12, 58]. The possible mechanisms include the following: (1) in the organ slice model of epilepsy induced by magnesium deficiency, the overexpression of RGMa can inhibit the N-methyl-D-aspartate receptor- (NMDAR-) mediated current, thereby inhibiting the overexcitation of hippocampal neurons [58–60]. (2) Lentiviral vector-induced RGMa overexpression in the hippocampus can inhibit seizures by inhibiting mossy fiber sprouting [12]. (3) Silencing miR-20a-5p, an upstream regulator of RGMa, inhibits neuronal branching and axon growth through the RGMa-RhoA pathway, thereby preventing epilepsy (Table 1) [25].

## 5. Summary

In conclusion, as an axon guidance molecule, RGMa widely participates in the development and pathological process of CNS to regulate cell proliferation, differentiation, adhesion, migration, neurogenesis, neural tube closure, neuronal apoptosis, synapse formation, growth cone collapse, axon growth inhibition, immune response, and neovascularization through RGMa-Neogenin, RGMa-BMPs, and other signaling pathways. Recent studies have found that RGMa can participate in the pathogenesis of MS, NMOSD, cerebral infarction, spinal cord injury, PD, epilepsy, and other CNS diseases. By regulating the expression of RGMa, it can reduce neural function damage and promote the recovery of neural function, indicating that RGMa may be a promising target molecule for the treatment of CNS diseases (Table 1). As the specific pathogenesis and signaling pathway of RGMa in CNS diseases are not fully clear, the randomized controlled clinical trials need to take years to conduct. Therefore, more in-depth analysis and large sample for randomized controlled clinical trials are required to elucidate the mechanism of RGMa in the guidance of clinical treatment of CNS diseases.

## Figures and Tables

**Table 1:** RGMa-related possible mechanisms involved in CNS diseases.

Disease	Expression site of RGMa	Possible mechanisms	Participants or models	Potential therapeutic target
MS	(1) Significantly upregulated in active and chronic MS lesions [22, 61] (2) Plasma RGMa is inversely related to delta EDSS [2, 6]	(1) Mediates immune responses [5, 37, 61–64] (2) Mediates CNS demyelination [38, 39] (3) Mediates neurodegeneration and inhibits neurite outgrowth [5, 7, 22, 40] (4) Inhibits neovascularization [5, 36, 65] (5) May be involved in BBB dysfunction [43–45, 66]	(1) MS patients [6, 22, 44] (2) EAE rats [5, 61]	(1) Targeting RGMa can improve functional recovery [22] (2) Anti-RGMa antibody can promote neurite outgrowth and remyelination [5, 22] (3) Anti-RGMa antibody can reduce immune responses [5]
NMOSD	Unknown	(1) May involve loss of AQP4, GFAP, and astrocytes [8, 46, 67] (2) May aggravate immune responses [8, 47, 68, 69] (3) May induce neuronal damage [8, 48, 49]	NMOSD model in rats [8]	Inhibition of RGMa can (1) delay onset [8] (2) relieve symptoms [8] (3) delay progression of NMOSD [8]
Ischemic stroke	Upregulated in vascular endothelium and neurons after I/R injury [9, 36]	(1) Inhibits axon growth by phosphorylating CRMP-2 [51] (2) Might inhibit angiogenesis by downregulating BDNF VEGF, Ang1, and Ang2 [35] (3) May reduce p-FAK (Tyr397) and VEGF via Neogenin and Unc5b [34] (4) Might impact LMC status [53] (5) May be involved in BBB dysfunction via the CDC-42/PAK-1 signal pathway [9]	(1) MCAO patients [53] (2) I/R injury model in rats [9, 35] (3) Endothelial cell in vitro [34]	(1) Anti-RGMa antibody or RGMa function-blocking peptide can significantly upregulate BDNF, VEGF, Ang1, and Ang2 [34, 35] (2) Inhibition of RGMa promotes functional recovery by promoting angiogenesis [35] (2) RGMa may predict LMC status [53] (3) Silencing RGMa ameliorates infarct volume, brain edema, and BBB dysfunction [9]
SCI	Upregulated around SCI lesion [30, 70]	(1) Inhibits neuronal survival [30] (2) Activates microglia [21]	(1) Patients with SCI [21] (2) SCI model in monkey [10, 71] (3) SCI model in mice [30, 72]	(1) Anti-RGMa antibody can promote axon regeneration, plasticity, motor recovery, and manual dexterity [10, 21, 30, 72] (2) Anti-RGMa antibody can relieve neuralgia [21]
PD	Significantly upregulated in the SN of patients with PD [11, 73]	(1) Induces selective degeneration of dopaminergic neurons in the SN [11, 74] (2) Activates microglia and astrocyte strongly [11, 74] (3) May inhibit neuronal survival by activating RhoA [58, 74]	(1) Patients with PD [11, 73] (2) RGMa can model PD in mouse [11]	(1) Inhibition of RGMa may modify PD [11, 75] (2) Regulating RGMa-Neogenin may promote cell replacement [74] (3) Anti-RGMa antibody may offer neuroprotection [74]
Seizures	Significantly decreased in epileptic patients and rat models [12, 58, 76]	(1) Partly via the FAK-p120Ras GAP-Ras signaling pathway suppresses MFS [12, 76] (3) Inhibits hyperexcitability of hippocampal neurons via suppressing NMDAR-mediated currents [58]	(1) Epileptic patients [58] (2) Pentylenetetrazol rat model [12] (3) Epileptic rat model [58] (4) Organotypic slice model [58]	Injection recombinant RGMa to intracerebroventricular or overexpression of RGMa suppresses MFS and seizures [12, 58, 76]

RGMa: repulsive guidance molecule-a; EAE: experimental autoimmune encephalomyelitis; NMOSD: neuromyelitis optica spectrum disorders; AQP4: aquaporin-4; MCAO: middle cerebral artery occlusion; I/R: ischemia-reperfusion; CRMP-2: collapsin response mediator protein 2; LMCs: leptomeningeal collaterals; SCI: spinal cord injury; PD: Parkinson's disease; SN: substantia nigra; PTZ: pentylenetetrazol; MFS: mossy fiber sprouting.
